# Supplementary material for: Viral inhibition of the anaphase promoting complex enhances replication by elevating nucleotide pools
Source: PLoS Pathog. 2026 Aug 12;22(8):e1013930. doi: 10.1371/journal.ppat.1013930 (PMC13524340; doi:10.1371/journal.ppat.1013930)
Supplement: Supplementary file 1 [file ppat.1013930.s011.pdf]

# Figure 1B

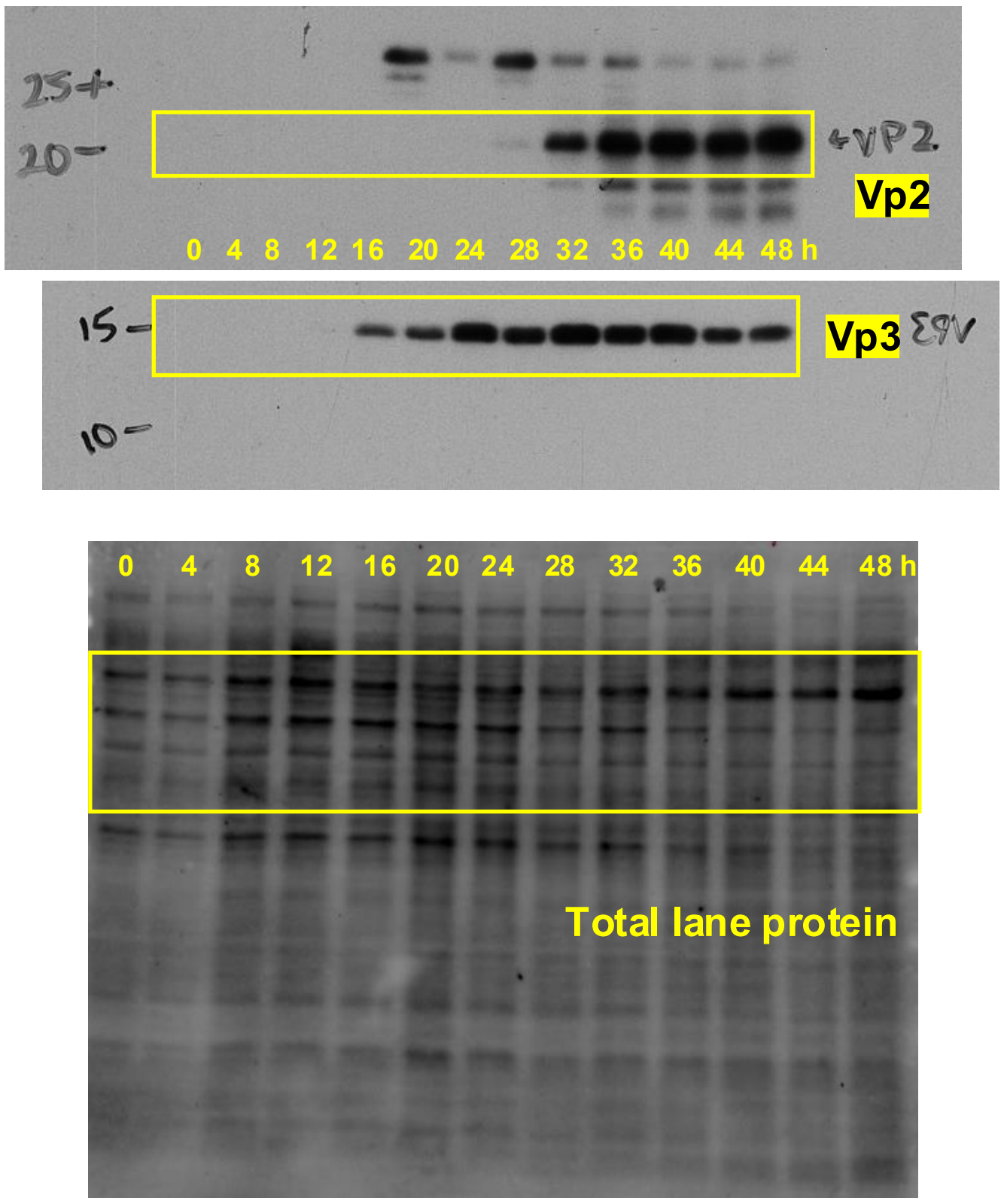


**Figure 1B**
**Vp3**: Perkin Elmer ECL, exposure on Autorad film
**Vp2**: Perkin Elmer ECL, exposure on Autorad film
**Total lane protein**: whole gel activation using the ChemiDoc XRS + stain-free enabled UV transillluminator from Bio-Rad

# Figure 2D

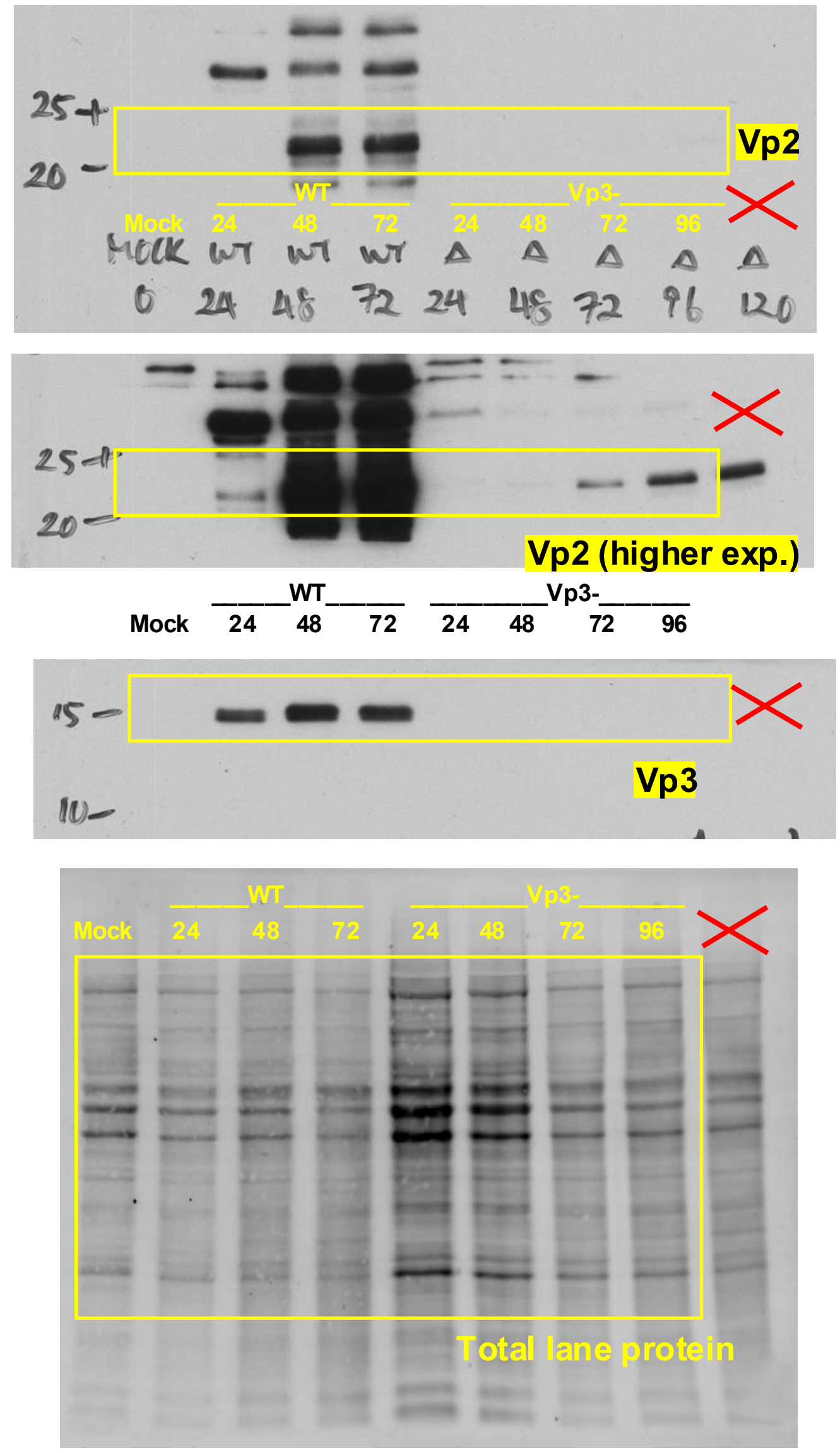


**Figure 2D**
**Vp2**: Perkin Elmer ECL, exposure on Autorad film
**Vp2 (higher exp.):** Same as Vp2, higher exposure
**Vp3**: Perkin Elmer ECL, exposure on Autorad film
**Total lane protein**: whole gel activation using the ChemiDoc XRS + stain-free enabled UV transillluminator from Bio-Rad

# Figure 4A

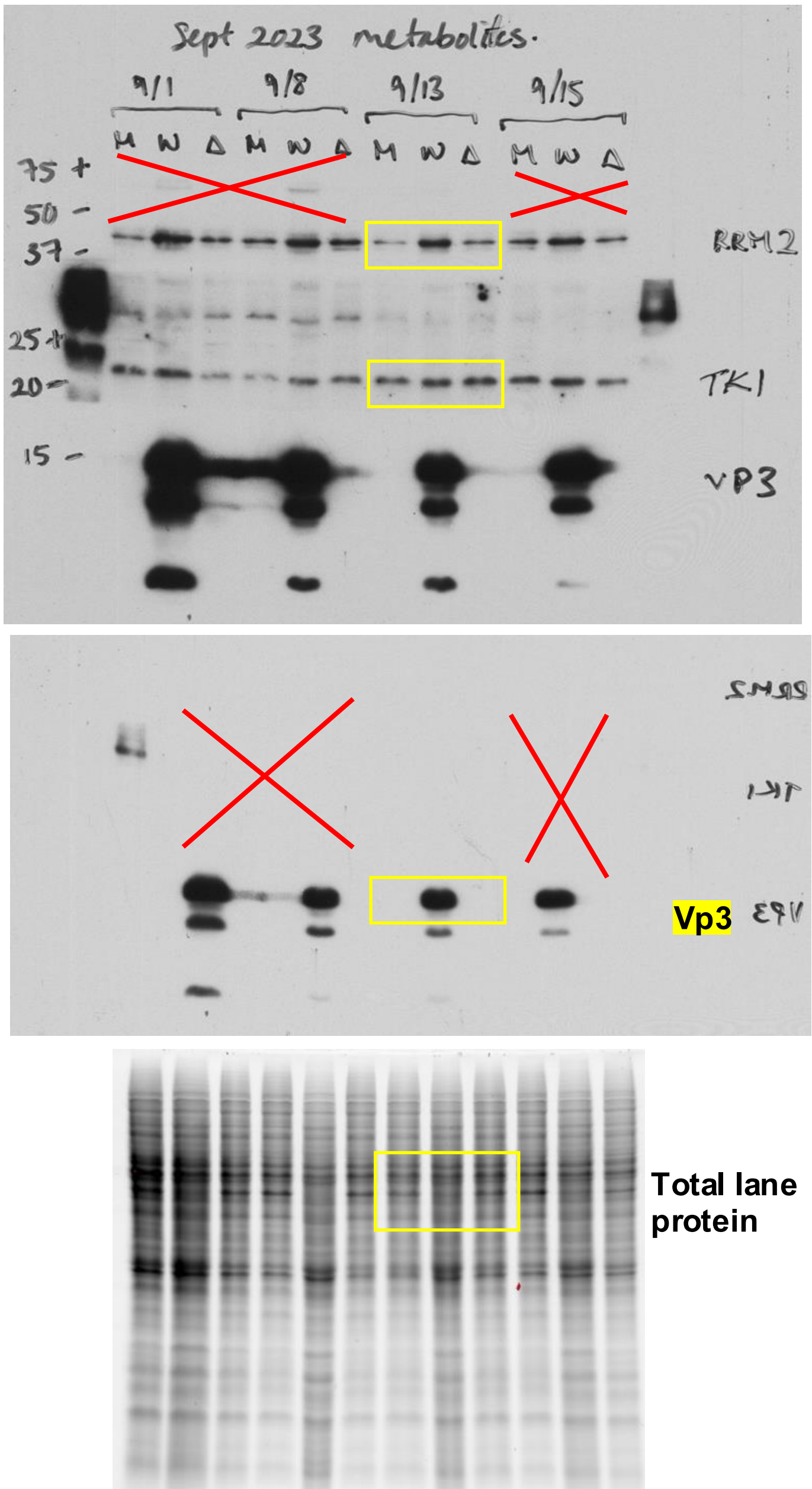


# Figure 4B

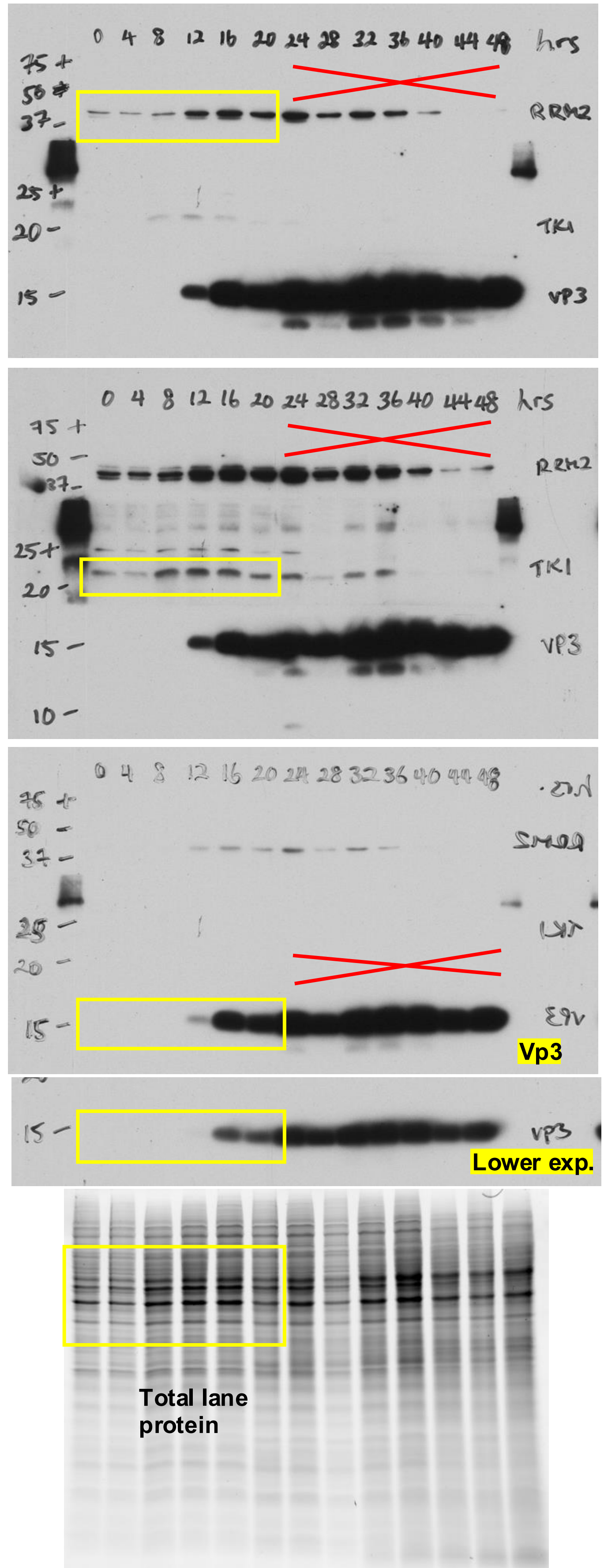


**Figure 4A**
**RRM2**: Clarity ECL, exposure on Autorad film
**TK1**: Clarity ECL, exposure on Autorad film
**Vp3**: Clarity ECL, exposure on Autorad film
**Total lane protein**: whole gel activation using the ChemiDoc XRS + stain-free enabled UV transilluminator from Bio-Rad

**Figure 4B**
**RRM2**: Clarity ECL, exposure on Autorad film
**TK1**: Clarity ECL, exposure on Autorad film
**Vp3**: Clarity ECL, exposure on Autorad film
**Vp3 (lower exp)**: Same as Vp3, lower exposure
**Total lane protein**: whole gel activation using the ChemiDoc XRS + stain-free enabled UV transilluminator from Bio-Rad

# Figure 4D

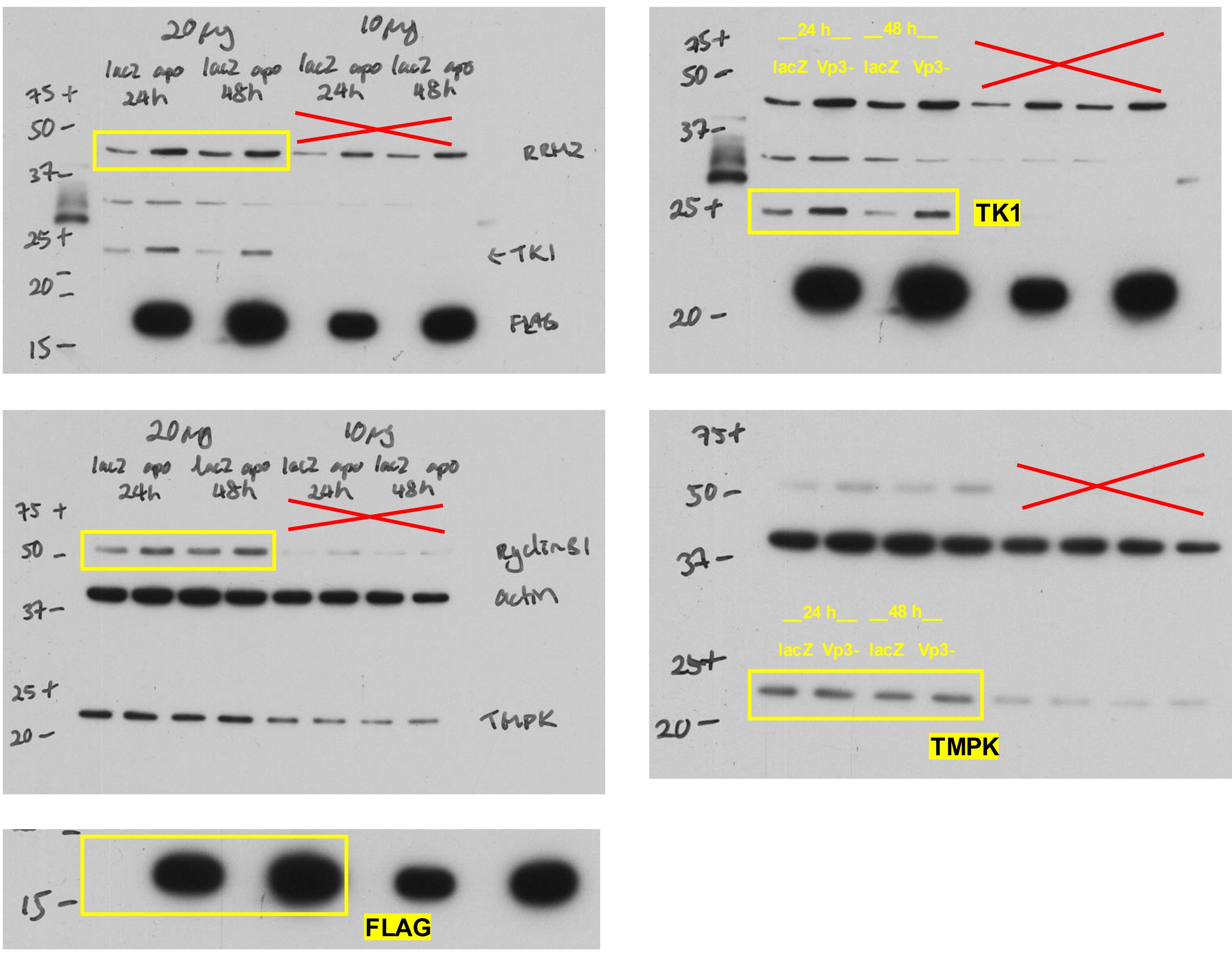


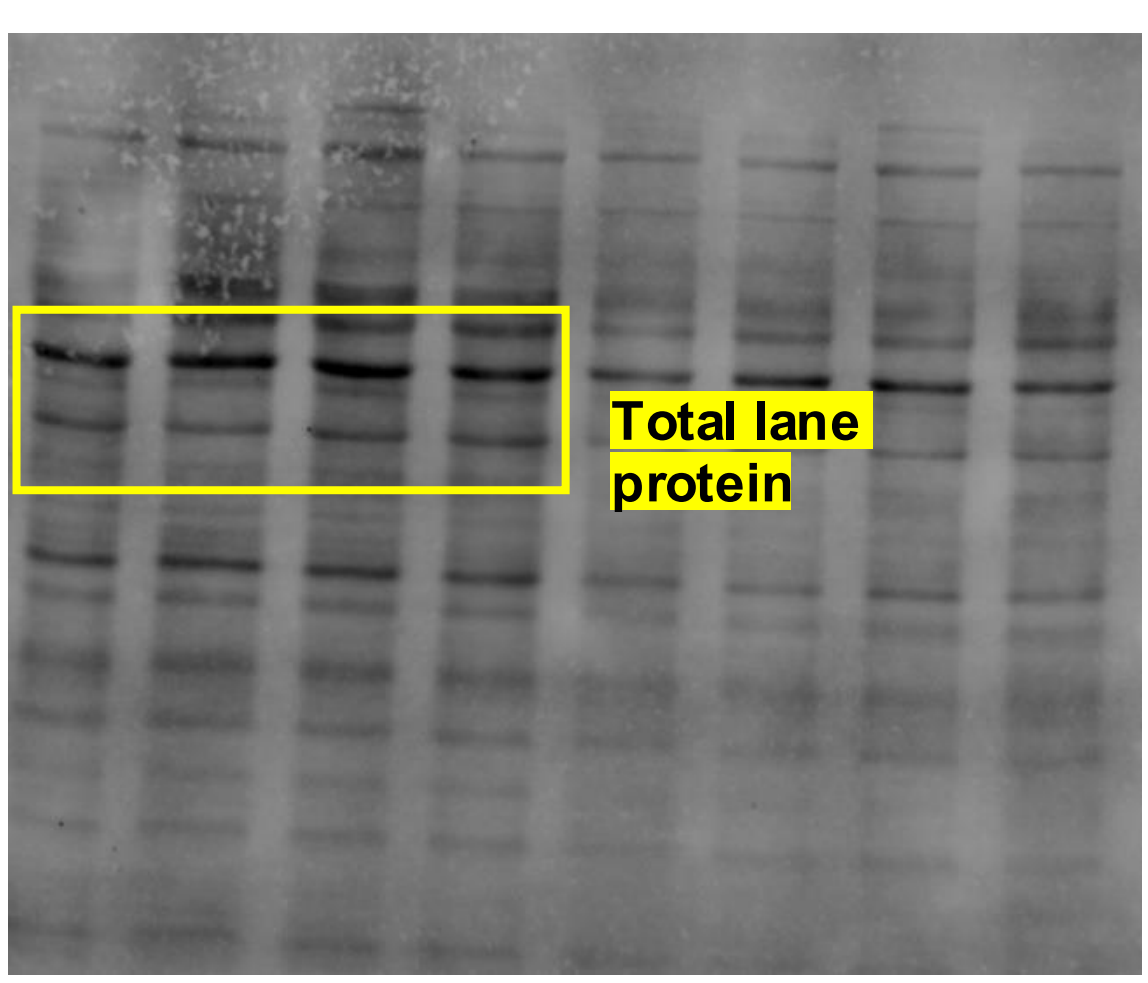


**Figure 4D**
**Cyclin B1**: Clarity ECL, exposure on Autorad film
**RRM2**: Clarity ECL, exposure on Autorad film
**TK1**: Clarity ECL, exposure on Autorad film
**TMPK**: Clarity ECL, exposure on Autorad film
**FLAG**: Clarity ECL, exposure on Autorad film
**Total lane protein**: whole gel activation using the ChemiDoc XRS + stain-free enabled UV transilluminator from Bio-Rad
