# Supplementary material for: Viral inhibition of the anaphase promoting complex enhances replication by elevating nucleotide pools
Source: PLoS Pathog. 2026 Aug 12;22(8):e1013930. doi: 10.1371/journal.ppat.1013930 (PMC13524340; doi:10.1371/journal.ppat.1013930)
Supplement: Supplementary file 2 [file ppat.1013930.s006.docx]

**S1 Fig. Cell viability of mock, CAV WT and CAV Vp3- infected MSB-1 cells quantified by the Zombie Red survival assay.** The results of this sensitive fluorescence-based survival assay are similar to those of the trypan blue staining assays, as presented in Figures 1D and 2F.
