# Supplementary material for: Viral inhibition of the anaphase promoting complex enhances replication by elevating nucleotide pools
Source: PLoS Pathog. 2026 Aug 12;22(8):e1013930. doi: 10.1371/journal.ppat.1013930 (PMC13524340; doi:10.1371/journal.ppat.1013930)
Supplement: Supplementary file 3 [file ppat.1013930.s007.docx]

**S2 Fig. Principal component analysis (PCA) plot of the metabolic profiles of mock, CAV WT and CAV Vp3- infected MSB-1 cells at 24 hours post-infection**.
