# Supplementary material for: Viral inhibition of the anaphase promoting complex enhances replication by elevating nucleotide pools
Source: PLoS Pathog. 2026 Aug 12;22(8):e1013930. doi: 10.1371/journal.ppat.1013930 (PMC13524340; doi:10.1371/journal.ppat.1013930)
Supplement: Supplementary file 4 [file ppat.1013930.s008.docx]

**S3 Fig. Normalized levels of nitrogenous bases compared across mock, CAV WT and CAV Vp3- infections.** *N* = 4; ns, not significant.
