# Supplementary material for: Viral inhibition of the anaphase promoting complex enhances replication by elevating nucleotide pools
Source: PLoS Pathog. 2026 Aug 12;22(8):e1013930. doi: 10.1371/journal.ppat.1013930 (PMC13524340; doi:10.1371/journal.ppat.1013930)
Supplement: Supplementary file 5 [file ppat.1013930.s009.docx]

**S4 Fig. mRNA transcript levels of different enzymes involved in nucleotide synthesis during CAV infection.**

Relative transcript levels of *RRM2*, *TK1* and *DTYMK* (TMPK) in cells infected with mock, CAV WT or CAV Vp3- at 12-hour timepoints were measured by RT-qPCR. Gene expression was normalized to that of reference genes *ACTB* (actin) and *GAPDH*. *ANAPC1* (APC1) and *ANAPC4* (APC4) were used as controls for comparison. Each gene at every timepoint was measured from three independent experiments. *N* = 3 for all groups. Statistical significance for each comparison: n.s. (not significant; *P* > 0.05).
