# Supplementary material for: Viral inhibition of the anaphase promoting complex enhances replication by elevating nucleotide pools
Source: PLoS Pathog. 2026 Aug 12;22(8):e1013930. doi: 10.1371/journal.ppat.1013930 (PMC13524340; doi:10.1371/journal.ppat.1013930)
Supplement: Supplementary file 6 [file ppat.1013930.s010.docx]

**S5 Fig. Heatmap visualizing the changes of the top 50 metabolites detected by LC-MS in H1299 cells infected with the Ad-lacZ and Ad-Vp3 adenoviral constructs, as well as mock infection.** Cells were harvested and metabolites were analysed at the 24- and 48-hour timepoints. Values are colour-coded on a blue-red gradient and are centred around 0, at which no relative change in levels is detected.
